# Supplementary material for: Resolving the full spectrum of human genome variation using Linked-Reads
Source: Genome Res. 2019 Apr;29(4):635–45. doi: 10.1101/gr.234443.118 (PMC6442396; doi:10.1101/gr.234443.118)
Supplement: Supplemental Material [file supp_gr.234443.118_Supplemental_longranger-2.2.zip › longranger-2.2.tar.bz2!/lr2.2-public/lib/stan/cmdstan/stan/doc/api/html/math_2matrix_2log__determinant_8hpp_source.html]

Stan: src/stan/math/matrix/log\_determinant.hpp Source File


|  |  |
| --- | --- |
|  | Stan  2.5.0  probability, sampling & optimization |


- Main Page
- Related Pages
- Namespaces
- Classes
- Files

- File List
- File Members

All Classes Namespaces Files Functions Variables Typedefs Enumerations Enumerator Friends Macros Pages

- src
- stan
- math
- matrix

log\_determinant.hpp

Go to the documentation of this file.

1 #ifndef STAN\_\_MATH\_\_MATRIX\_\_LOG\_DETERMINANT\_HPP

2 #define STAN\_\_MATH\_\_MATRIX\_\_LOG\_DETERMINANT\_HPP

3

4 #include <stan/math/matrix/Eigen.hpp>

5 #include <stan/math/error\_handling/matrix/check\_square.hpp>

6

7 namespace stan {

8  namespace math {

9

17  template <typename T,int R, int C>

18  inline T log\_determinant(const Eigen::Matrix<T,R,C>& m) {

19  stan::math::check\_square("log\_determinant(%1%)",m,"m",(double\*)0);

20  return m.colPivHouseholderQr().logAbsDeterminant();

21  }

22

23  }

24 }

25 #endif

Eigen.hpp

stan::math::log\_determinant

T log\_determinant(const Eigen::Matrix< T, R, C > &m)

Returns the log absolute determinant of the specified square matrix.

**Definition:** log\_determinant.hpp:18

stan::math::check\_square

bool check\_square(const char \*function, const Eigen::Matrix< T\_y, Eigen::Dynamic, Eigen::Dynamic > &y, const char \*name, T\_result \*result)

Return true if the specified matrix is square.

**Definition:** check\_square.hpp:24

check\_square.hpp


---

[ Stan Home Page ]

*© 2011–2014,
Stan Development Team.*
